# Integrative Transcriptomics Reveals Activation of Innate Immune Responses and Inhibition of Inflammation During Oral Immunotherapy for Egg Allergy in Children

**DOI:** 10.3389/fimmu.2021.704633

**Published:** 2021-12-15

**Authors:** Piia Karisola, Kati Palosuo, Victoria Hinkkanen, Lukas Wisgrill, Terhi Savinko, Nanna Fyhrquist, Harri Alenius, Mika J. Mäkelä

**Affiliations:** ^1^ Human Microbiome (HUMI) Research Program, Medical Faculty, University of Helsinki, Helsinki, Finland; ^2^ Skin and Allergy Hospital, Helsinki University Hospital, University of Helsinki, Helsinki, Finland; ^3^ Division of Neonatology, Pediatric Intensive Care and Neuropediatrics, Comprehensive Center for Pediatrics, Medical University of Vienna, Vienna, Austria; ^4^ Institute of Environmental Medicine (IMM), Karolinska Institutet, Stockholm, Sweden

**Keywords:** food allergy, oral immunotherapy, microarray, desensitization, inflammation

## Abstract

We previously reported the results of a randomized, open-label trial of egg oral immunotherapy (OIT) in 50 children where 44% were desensitized and 46% were partially desensitized after 8 months of treatment. Here we focus on cell-mediated molecular mechanisms driving desensitization during egg OIT. We sought to determine whether changes in genome-wide gene expression in blood cells during egg OIT correlate with humoral responses and the clinical outcome. The blood cell transcriptome of 50 children receiving egg OIT was profiled using peripheral blood mononuclear cell (PBMC) samples obtained at baseline and after 3 and 8 months of OIT. We identified 467 differentially expressed genes (DEGs) after 3 or 8 months of egg OIT. At 8 months, 86% of the DEGs were downregulated and played a role in the signaling of TREM1, IL-6, and IL-17. In correlation analyses, Gal d 1–4-specific IgG4 antibodies associated positively with DEGs playing a role in pathogen recognition and antigen presentation and negatively with DEGs playing a role in the signaling of IL-10, IL-6, and IL-17. Desensitized and partially desensitized patients had differences in their antibody responses, and although most of the transcriptomic changes were shared, both groups had also specific patterns, which suggest slower changes in partially desensitized and activation of NK cells in the desensitized group. OIT for egg allergy in children inhibits inflammation and activates innate immune responses regardless of the clinical outcome at 8 months. Changes in gene expression patterns first appear as posttranslational protein modifications, followed by more sustained epigenetic gene regulatory functions related to successful desensitization.

## Introduction

Oral immunotherapy (OIT) is still an experimental therapeutic approach for food allergy, although new strategies have been developed, and it is introduced in clinical practices in selected medical centers during the recent years (1). Ingestion of gradually increasing doses of specific food allergens renders an individual temporarily less reactive to the allergen (desensitization), and if continued, it may eventually result in long-lasting changes (sustained unresponsiveness and tolerance). OIT can successfully desensitize up to 80% of children with persistent milk, egg, wheat, or peanut allergy (2–4). However, adverse events are frequent, and severe allergic reactions during OIT have been reported. Some patients require prolonged treatment protocols, and 20–40% fail to respond (2, 5). Open questions remain regarding optimal desensitization protocols, safety, and the selection of patients responding favorably to OIT. Early biomarkers and predictors for the success of OIT would be of great clinical relevance.

Although the exact mechanisms mediating desensitization and long-lasting tolerance in OIT are unknown, some of the underlying immunologic events have been elucidated. Mast cell and basophil degranulation is decreased already at the early stages of OIT, and allergen-specific Th2 cell responses are attenuated as a key event leading to sustained unresponsiveness and tolerance (6). These changes are accompanied by the increased production of allergen-specific IgG4, followed by decreased IgE production (7–9). Ultimately, the number of effector cells in target tissues is decreased, accounting for clinical antigen hyporesponsiveness. It remains unclear how food OIT modulates gene expression at the individual level. Several studies have associated susceptibility genes with food allergy, but little emphasis has been given on characterizing gene expression profiles that result from OIT for food allergy (10).

In our previous study, we described the results of a randomized, open-label trial of egg OIT in 50 children where 44% were desensitized and 46% were partially desensitized after 8 months of treatment (11). We defined desensitization as the ability to consume 1 g of egg white protein and partial desensitization as the ability to consume any dose below 1 g of egg white protein without symptoms. We showed that high baseline egg white-specific IgE levels and polysensitization to the egg allergen molecules Gal d 1–4 were related with discontinuation and the need for individually adjusted, prolonged treatment protocols.

In this study, we focused on the cellular mechanisms driving desensitization during egg OIT. To the best of our knowledge, this is the first and the largest follow-up study on PBMC transcriptomics of food-allergic patients receiving OIT. We aimed to determine whether changes in genome-wide gene expression after 0, 3, and 8 months of OIT correlate with humoral responses and the clinical outcome. We also examined how egg OIT modifies allergic inflammation and humoral responses. Using these data, we integrated results from humoral mediators (antibodies and cytokines) and advanced gene expression analysis to identify key genes, biological processes, and cell types involved in allergen desensitization during OIT. Our results may facilitate the development of potential biomarkers for predicting the outcome of OIT as well as the planning of individually adjusted, personalized treatment protocols.

## Methods

### Study Population

The study included 50 children and adolescents from the Department of Allergology, Helsinki University Hospital, Finland, aged 6–17 years, with egg allergy, diagnosed by double-blind, placebo-controlled food challenge to heated egg (12). The inclusion and exclusion criteria are reported in our previous study (11). The Helsinki University Hospital of Children and Adolescents Ethics Committee approved the study, and each participant above 6 years of age as well as his/her guardian gave written informed consent.

### OIT Protocol and Collection of Blood Samples

OIT was carried out as previously described (11) with pasteurized, spray-dried, raw egg white powder (Dava Foods, Piispanristi, Finland) with daily dosing at home. The dose was increased weekly for the first 3 weeks and from then on biweekly. The build-up phase lasted for 8 months. The target maintenance dose was 1 g of egg white protein, corresponding approximately to one-third of the protein content of an egg white. Venous blood samples were taken at baseline and after 3 and 8 months of OIT.

### Peripheral Blood Mononuclear Cells

Peripheral blood mononuclear cells (PBMCs) were isolated from 8 ml of whole blood by a CPT tube system (BD Sciences). The separated plasma was aliquoted and stored at -20°C. The extracted PBMCs were frozen in cell-freezing medium (Gibco) and stored in a deep freezer (-80°C). After thawing the samples, the number of total cells was counted (Beckman Coulter AcT/Diff), and the relative proportions of cell populations were determined by flow cytometry using surface markers. Subsets of T cells (CD3^+^), B cells (CD19^+^), NK cells (CD16^+^ CD56^+^), and monocytes (CD14^+^) were identified. In addition, cell viability was checked, and the percentage of dead cells was determined.

### Leukocyte Deconvolution Analysis—CIBERSORT

The CIBERSORT (cibersort.stanford.edu) algorithm was used to estimate the leukocyte subset proportions of 22 individual immune cells utilizing the “L22” validated gene signature matrix (13). The estimations are based on 1,000 permutations. No significance filter has been applied to the estimated cell fractions to include all samples for further analysis.

### Humoral Analyses

Changes in levels of specific IgE, IgG4, and IgA antibodies to the major egg allergens (Gal d1–d4) were measured by ImmunoCAP (Thermo Fisher, Uppsala, Sweden) and cytokines by Luminex Bio-Plex system (Bio-Rad).

### Gene Expression Arrays and Associated Analyses

Total RNA was isolated from 2 × 10^6^ PBMCs by AllPrep Plus (Qiagen). The quantity and quality of RNA was measured by NanoDrop/Qubit and BioAnalyzer, respectively. Upon extraction, 100 μg of good-quality (RIN > 8) total RNA from PBMCs was used for microarrays. The genome-wide expression of PBMCs was studied on Agilent SurePrint G3 Human Gene Expression v3 arrays at 0, 3, and 8 months of OIT. The RNA was primed with oligo-dT (T7) promoter primers and converted to cDNA with AffinityScript RNase Block (Low Input Quick Amp Labeling Kit, Two-color, Agilent Technologies), which was labeled with Cy3 and Cy5 dyes (Agilent Technologies) and amplified using T7 RNA polymerase amplification method (Low Input Quick Amp Labeling Kit, Agilent Technologies) according to the instructions of the manufacturer. The amplified samples were cleaned up with Qiagen’s RNeasy mini kit (Qiagen, GmbH, Hilden, Germany). The primed cDNAs were hybridized for 17 h at +65°C to the two-color 60-mer oligo microarray slides (Agilent Technologies). After washing and scanning the slides with Agilent SureScan G2505C (Agilent Technologies), raw intensity values were obtained with the Feature Extraction software (version 11.5.11, Agilent Technologies).

Data was imported to eUtopia (14) software, and the median intensities were log2-transformed and quantile-normalized with Bioconductor package Limma (15). The SVA package with Combat function was utilized for the removal of batch effects emerging from labeling and array variance (16). Linear model was fitted to the batch-corrected data, and pairwise comparisons were performed by empirical Bayes method. Genes with linear fold change ≥|1.5| or ≥|1.3| (for Part/Des comparisons) and with Benjamini–Hochberg adjusted *P*-value ≤0.05 were considered significantly differentially expressed when compared to samples of 0 months. Hierarchical clustering of differentially expressed genes was done within the Perseus data analysis platform (17). Differentially expressed genes (DEGs) from different timepoints were independently Z-score-normalized. The Euclidean method and the k-means algorithm were respectively used to determine the distance between two dataset points and to define the distance between two clusters. A hierarchical cluster of the top DEGs (based on adjusted *p*-values) are depicted in the form of heat maps for each timepoint. Upregulated genes (red) are those genes that have a positive standard deviation from the mean expression of the genes across all exposures and vice versa for downregulated genes (blue). To evaluate the distribution of differentially expressed genes between specified contrast sets, Venn diagrams were created using Venny (18). The microarray data have been deposited in NCBI Expression Omnibus (GEO) database and are accessible through GEO Series accession number GSE178460.

The most relevant gene products related to OIT were found from the DEGs at different timepoints by analyzing their biological functions, signaling pathways, and upstream gene regions in pathway-oriented programs Ingenuity Pathways Analysis (IPA) and Gene Ontology Classification (Panther).

### Correlation Analysis

To study the interactions between the differentially expressed genes and antibody responses, we performed Pearson’s correlation and associated heat maps with Perseus program. We correlated the patient-specific antibody concentrations to patient-specific gene expression intensities of all the 467 DEGs. All timepoints (0, 3, and 8 months) were included in the analysis. Using threshold of correlation coefficient *r* higher than |0.35| and *P*-value less than 0.00001 to at least one antibody concentration, we selected the most significant genes (270 DEGs) and made a Euclidean heat map with gene clusters.

### Dose–Regression Analysis

The egg protein dose (20–1,000 mg) of individual patients reached after 8 months of OIT was used for regression analyses. The dose was compared with either gene expression at 8 months (330 DEGs) or with cytokine or antibody concentration or blood cellular compositions at the beginning (0 months) or after 8 months of OIT. Variables, with a significantly non-zero slope and regression *P*-value <0.05, were selected for correlation analyses. The Pearson correlations and *q* values were calculated by GraphPad Prism.

## Results

### Desensitization

The clinical OIT outcome is reported in detail in our previous study (11). After 8 months of OIT, 22/50 patients (44%) reached the maintenance dose of 1 g of egg white protein (desensitized) and 23/50 (46%) reached a dose less than 1 g (partially desensitized), with a median (IQR) dose of 300 (175–700) mg and a range of 20–700 mg. Five patients (10%) discontinued during the build-up phase.

### Kinetics of Immunoglobulins and Cytokines During OIT

**Figure 1:**
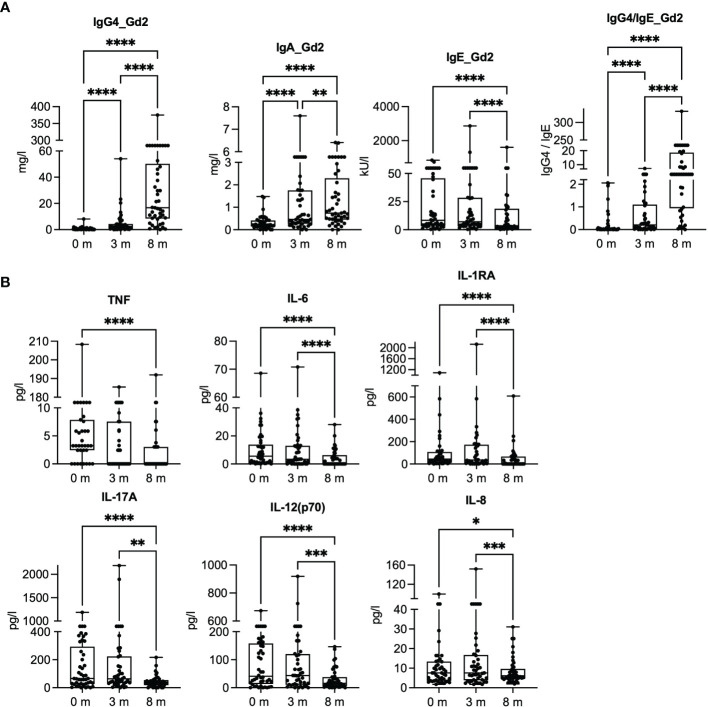
Gal d 2-specific antibody responses and inflammatory mediators in plasma at baseline (0 months) and after 3 and 8 months of oral immunotherapy. **(A)** Concentrations of Gal d 2-specific IgG4, IgA, and IgE antibodies were measured by ImmunoCAP (*n* = 45). **(B)** Inflammatory mediator concentrations were measured in plasma by Luminex at different timepoints (*n* = 43–45/timepoint). The results are expressed as mean with SEM. Significance is indicated by *p < 0.05, **p < 0.01, ***p < 0.001, and ****p < 0.0001 determined by ANOVA, Friedman test with Dunn’s *post-hoc* correction **(A)**, and mixed-effect analysis with Tukey’s multiple-comparison test **(B)**.

We measured the levels of plasma Gal d 2-specific IgA, IgG4, and IgE antibodies and several cytokines during the 8 months of dose escalation phase (**Figure 1**). The concentrations of Gal d 2-specific IgG4 and IgA antibodies increased linearly, while IgE increased during the first 3 months, followed by a decrease at 8 months (**Figure 1A**). The antibody responses to Gal d 1, 3, and 4 showed a similar shifting during OIT (11). The concentration of tumor necrosis factor (TNF) and IL-6 decreased, particularly at 8 months, whereas the concentrations of soluble IL-1 receptor accessor (IL-1RA) protein, IL-17A, IL-12p70, and IL-8 first slightly increased and then decreased by 8 months (**Figure 1B**).

### OIT Modifies Blood Cell Composition

**Figure 2:**
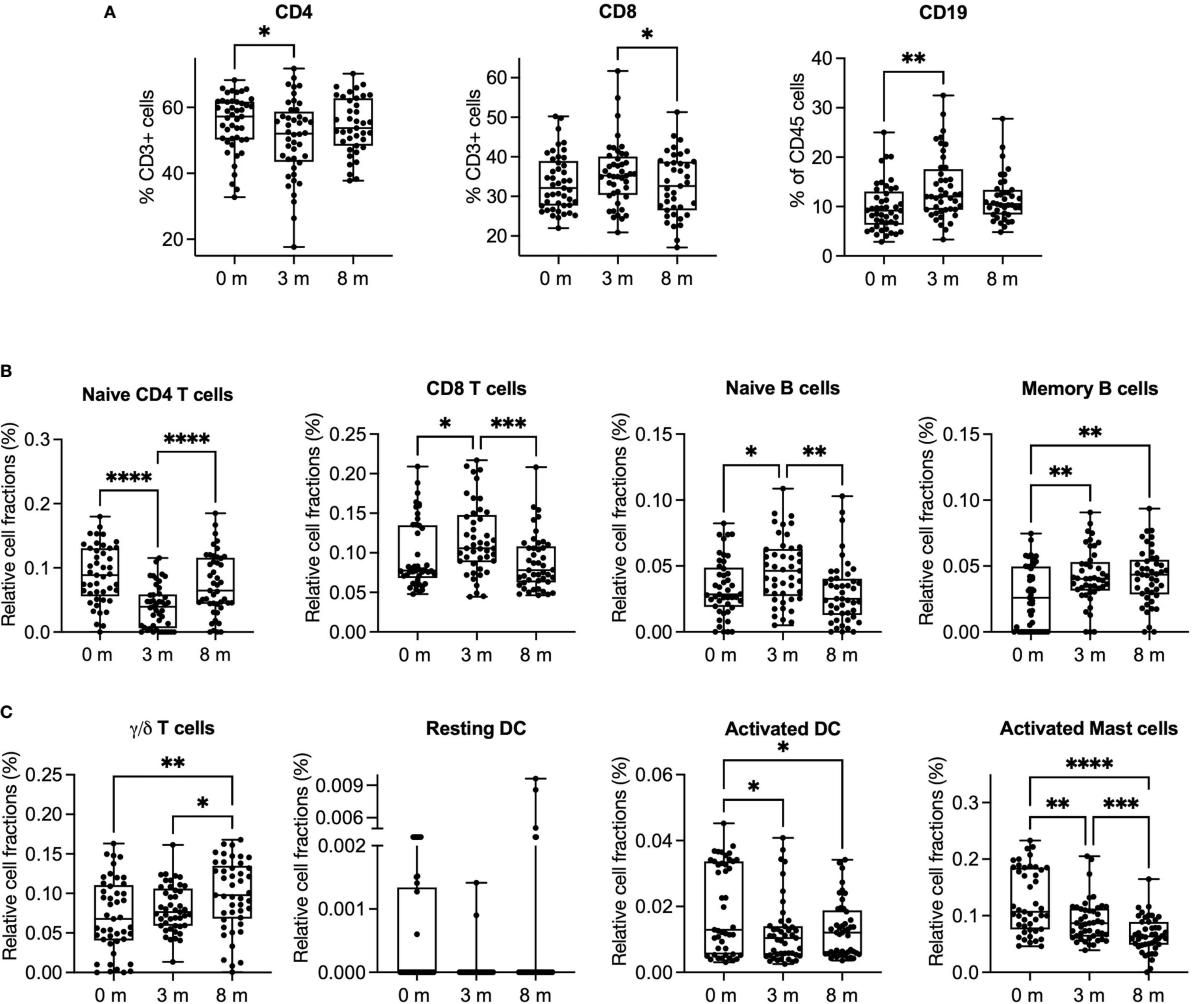
Cell distribution in peripheral blood mononuclear cells and their cellular deconvolution. **(A)** The percentage of CD4^+^ and CD8^+^ T cells from all T cells (CD3^+^) and the percentage of B cells (CD19^+^) from all leukocytes (CD45^+^) were measured by flow cytometry. All points and the mean are shown. Significance is determined by mixed-effect analysis with Tukey’s multiple-comparison test, *n* = 39–45. **(B)** The ratios of CD4 and CD8 T cells and B cells were estimated by cell deconvolution method. **(C)** The ratios of gamma/delta (γ/δ) T cells, activated/resting dendritic cells, and activated mast cells were estimated by deconvolution method. Differences in estimated cell fractions between different timepoints were analyzed using the repeated-measures ANOVA with Tukey’s multiple-comparison test (*n* = 45/timepoint). Significance is indicated by *p < 0.05, **p < 0.01, ***p < 0.001, and ****p < 0.0001.

To identify the effects of OIT on blood cell composition, we phenotyped the PBMCs by flow cytometry (**Figure 2A**) and used cell deconvolution method to identify transcriptional cell subtype signatures (**Figures 2B, C**). After 3 months of OIT, the number of CD4^+^ T cells decreased, and the number of CD8^+^ T cells and B cells (CD19^+^) increased. By 8 months of OIT, the levels of CD4^+^ and CD8^+^ T cells returned close to baseline values, while the levels of memory B cells remained increased. By utilizing the cell deconvolution method, we estimated that the number of γ/δ T cells increased, while the number of resting and activated dendritic cells (DC) and activated mast cells decreased during the OIT (**Figure 2C**). Although the flow cytometry analysis did not reach statistical significance, the trend of CD4, CD8, and CD19 followed the results obtained by deconvolution method.

### Analysis of Differentially Expressed Genes

**Figure 3:**
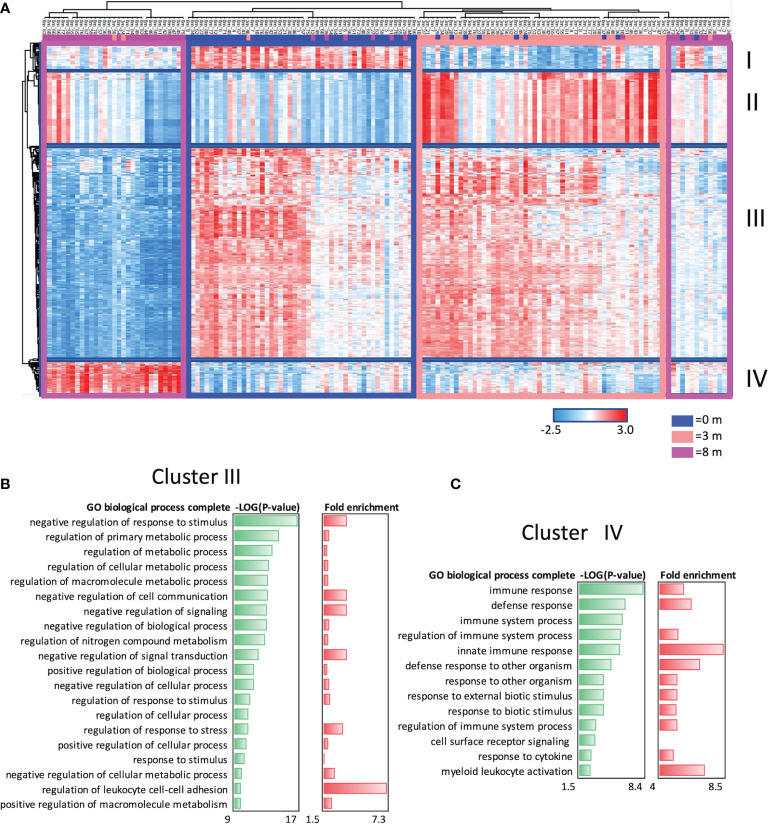
Differentially expressed genes (DEGs) at baseline and after 3 and 8 months of oral immunotherapy. **(A)** Heat map of 467 ANOVA-significant DEGs, which form four (I–IV) gene clusters at different timepoints. **(B)** Cluster III enriches the regulation of metabolic processes, and **(C)** cluster IV enriches the immune responses in Gene Ontology biological processes analyzed with Panther classification system. Clusters I and II did not enrich any known pathways. Overrepresentation tests were performed using Fisher’s exact *t*-test and Bonferroni’s correction in the Panther.

Genome-wide gene expression arrays were used to study the transcriptome of the PBMCs at baseline (0 months) and after 3 and 8 months of OIT (**Figure 3**). After data normalization and removal of technical batch effects, we identified the DEGs by comparing gene expressions at 3 or 8 months to baseline (0 months). The samples were clustered based on their collection time (0, 3, or 8 months) (**Figure 3A**). The DEGs formed four gene function-related clusters, of which clusters I and II showed no known functions, cluster III was associated with regulation of metabolic processes (**Figure 3B**), and cluster IV was enriched to immune and defense responses in the biological processes of Gene Ontology (GO) (**Figure 3C**).

**Figure 4:**
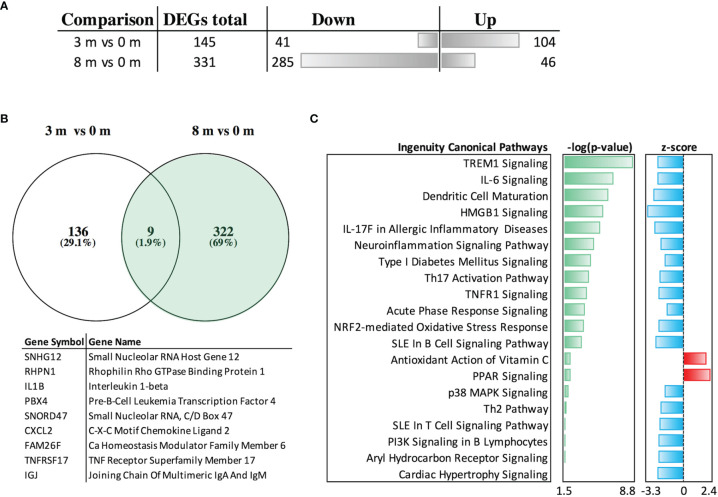
Differentially expressed genes (DEGs) at different timepoints, their overlap in the VENN diagram, and function of oral immunotherapy-induced genes at 3 and 8 months *vs*. those at 0 months. In **(A)**, the total number and directionality of the 467 DEGs shown at 3 and 8 months are compared to those at baseline (0 months). The threshold values were selected for *P*-value ≤0.05 and for linear fold change ≥|1.5|. **(B)** Overlapping of DEGs is shown in the VENN diagram with nine overlapping gene symbols and gene names. **(C)** Canonical pathway analysis of the gene expression at 8 months *vs*. that at 0 months by Ingenuity pathway analysis using threshold values [-log(*P*-value) ≥ 1.5 and absolute z-score value ≥1.5].

We identified 476 DEGs, 145 of which were specific for 3 months (3 *vs*. 0 months) and 331 for 8 months of OIT (8 *vs*. 0 months) (**Figure 4A** and lists of DEGs in **Supplementary Table S1**). After 3 months of OIT, 104/145 DEGs (72%) were upregulated, whereas after 8 months, 285/331 DEGs (86%) were downregulated (**Figure 4A**). VENN diagram was used to compare the distribution of common and unique DEGs (**Figure 4B**). The nine DEGs common for both timepoints included small nucleolar RNAs, IL-1β, and tumor necrosis factor receptor superfamily member 17, also known as a B-cell maturation antigen (**Figure 4B**).

The DEGs specific for 3 months were mostly long non-coding gene products with unknown functions and did not enrich any known pathways. The 331 DEGs specific for 8 months (8 *vs*. 0 months) showed enrichment to inflammatory mediators (IL-6, IL-17, and TNF), Th2 pathway, and associated gene regulators [triggering receptor expressed on myeloid cells (TREM), HMGB1, and AHR] in canonical IPA (**Figure 4C**). When the categories of diseases and biological functions were studied in IPA, the DEGs that were the most downregulated enriched to cell migration (especially leukocytes and neutrophils), leukopoiesis, and differentiation of mononuclear leukocytes (**Supplementary Figure S1**).

### Desensitized and Partially Desensitized Subjects

In order to study whether there are differences between the desensitized (Des) and partially (Part) desensitized patients, we studied their responses in antibody production (**Supplementary Figure S2**), cytokine responses (**Supplementary Figure S3A**), and PBMC cell content (**Supplementary Figure S3B**). The antibody patterns looked similar, and in most of the cases, the antibody production was more intense in partially desensitized patients (**Supplementary Figure S2**). Especially IgG4 is increased during the OIT in both groups. Only IgE to Gal d2 and Gal d3 was significantly increased in Part *vs*. Des at 8 months, and IgA to Gal d2 was significantly increased in Part when compared to Des at 3 months after the beginning of the OIT (**Supplementary Figure S2**). At all other cases, the Part did not differ from the Des group (**Supplementary Figure S2**). The cytokine patterns looked also similar, and there are no statistically different significances between the Part and the Des groups, although the TNF and IL-6 productions are significantly decreased within the Part group (**Supplementary Figure S3A**). No statistical differences were identified in the cellular composition of PBMCs within different clinical outcomes (Part *vs*. Des) (**Supplementary Figure S3B**).

### Correlation of Gal d 1–4-Specific IgE, IgG4, and IgA Antibodies to DEGs

**Figure 5:**
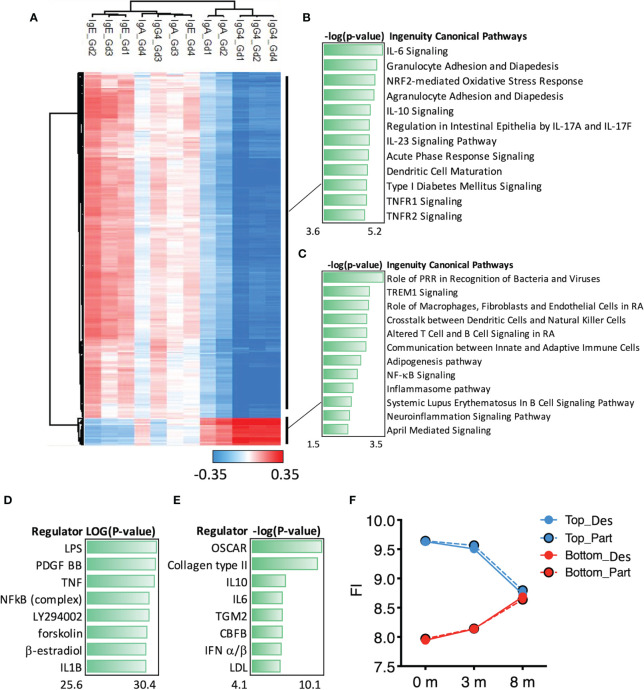
Correlation of Gal d 1–4-specific antibodies to differentially expressed genes (DEGs) (8 *vs*. 0 months). **(A)** Correlation analysis of all the 467 DEGs was correlated patient-specifically to their concentrations of allergen-specific IgE, IgG4, and IgA antibodies at timepoints 0, 3, and 8 months (*n* = 45/group). Pearson correlations with *r* > |0.35| are shown in the heat map (all DEGs have Pearson *P*-value <0.00001). The function of **(B)** the top (250 DEGs) and **(C)** the bottom (20 DEGs) gene clusters was studied by canonical pathway analysis (IPA). Upstream Regulators analysis was used to study the most significant gene regulators in **(D)** the top and in **(E)** the bottom gene clusters. **(F)** Mean changes of gene expression (as Log2-transformed fold changes) in the top cluster (250 DEGs) and the bottom cluster (20 DEGs) were followed at different timepoints based on the desensitization outcome in egg-allergic children: desensitized (Des, *n* = 22) or partially desensitized (Part, *n* = 23).

To link the changes in gene expression and humoral responses, we correlated the 467 DEGs to Gal d 1–4-specific IgE, IgG4, and IgA concentrations at all timepoints (**Figure 5**). This yielded two antibody–gene correlation clusters (**Figure 5A**). The upper cluster of genes (250 DEGs) associated mostly negatively with IgG4 antibodies to Gal d 1, 2, and 4 and IgA antibodies to Gal d 1 and 2, while the lower cluster of genes (20 DEGs) associated positively with the same antibodies (**Figure 5A**).

The upper gene cluster plays a role in granulocyte adhesion and diapedesis, signaling of IL-6, IL-10, and IL-17A according to canonical pathways in IPA (**Figure 5B**). These pathways are regulated by lipopolysaccharide (LPS), platelet-derived growth factor subunit B, and TNF (**Figure 5D**). The lower gene cluster participates in innate immunity through pattern recognition receptors (PRRs) and TREMs (**Figure 5C**). These genes are regulated by an osteoclast-associated receptor, IL-10, and interferons α, β, and γ (IFN-α/β/γ) (**Figure 5E**). There were no differences in the expression levels of the upper and lower gene clusters in the desensitized and partially desensitized subjects (**Figure 5F**).

### Desensitized and Partially Desensitized Subjects Have Different Gene Expressions at 3 and 8 Months After the OIT

**Figure 6:**
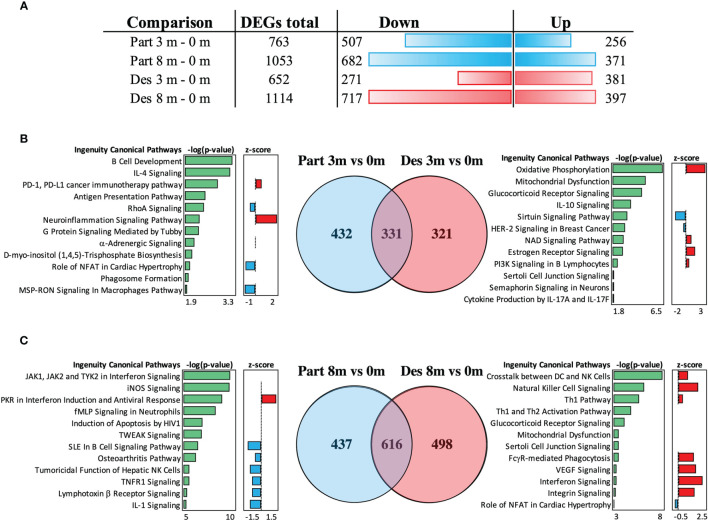
Differential gene expression analysis of peripheral blood mononuclear cells extracted from desensitized (Des) and partially (Part) desensitized patients. **(A)** The total number and directionality of the differentially expressed genes (DEGs) is shown at 3 and 8 months compared to baseline (0 months). The threshold values were selected for Benjamini–Hochberg adjusted *P*-value ≤0.05 and for linear fold change ≥|1.3|. **(B)** Overlap of DEGs at 3 *vs*. 0 months is shown in the VENN diagram and their function in canonical pathway analysis (IPA). **(C)** Overlap of DEGs at 3 *vs*. 0 months is shown in VENN diagram and their function in Canonical pathway analysis (IPA). The significance values (-log of the *p*-value of overlap) for the canonical pathways are calculated by right-tailed Fisher’s exact test. The top 12 pathways and their predicted activation or inhibition (z-scores) are shown. Des, *n* = 22; Part, *n* = 23).

The gene expression of desensitized (Des) and partially (Part) desensitized patients was followed after 3 and 8 months after the OIT within these groups, which were identified by clinical outcome on egg ingestion tolerance. The Part and Des groups had 763 and 652 DEGs after 3 months of OIT, and after 8 months of OIT, they respectively had 1,053 and 1,114 DEGs, when compared to gene expression at 0 months and analyzed with thresholds of *P*-value ≤0.05 and linear fold change ≥|1.3| (**Figure 6A**). These Part and Des DEGs formed three sample clusters, mostly based on the sample collection time (**Supplementary Figure S4**). The number of overlapping DEGs was studied in a VENN diagram, and about half of the genes (331 DEGs) and 2/3 (616 DEGs) of the group-specific DEGs were shared between the groups at 3 and at 8 months, respectively (**Figures 6B, C**). At 3 months, the common genes were involved in antigen presentation, B cell development, and IL-4 signaling, and at 8 months, the shared genes played a role in sirtuin and GADD45 signaling (**Supplementary Figure S5**). At 3 months, the DEGs of Part patients enriched to pathways participating in B cell development, IL-4 signaling, and antigen presentation, whereas the DEGs of Des patients enriched to oxidative phosphorylation, glucocorticoid receptor, and IL-10 signaling (**Supplementary Figure 6B**). At 8 months, the DEGs of Part patients enriched to pathways which were involved in interferon and iNOS signaling and antiviral responses, while the DEGs of Des patients enriched to a crosstalk between DC and NK cells, NK cell signaling, and Th1/Th2 pathways (**Figure 6C**).

### Prediction of OIT Effectiveness by Dose Regression

To identify factors influencing the OIT outcome, we performed dose regression analyses (**Supplementary Figure S6**). We compared gene expression (DEGs), cytokine production, antibody production, or blood cellular composition at the beginning (0 months) or after 8 months of OIT, with the amount of egg protein (20–1,000 mg) tolerated in the Des and the Part patients. Factors with a significantly non-zero slope and regression *P*-value <0.05 were selected for correlation analyses. After calculating the adjusted *P*-values for Pearson correlation to OIT dose at 8 months, only IgE concentrations of Gal d1–d4 reached statistical significance at both 0 and 8 months and IgA to Gal d2 at 8 months (**Supplementary Figures S6A, B**).

## Discussion

Oral immunotherapy is an experimental treatment for food allergy, which aims to increase the threshold amount of food allergen tolerated without symptoms. The exact molecular mechanisms mediating desensitization and the development of tolerance to foods during OIT are largely unknown. Our previous study (11) describes an effective OIT protocol of the egg of a hen using raw, pasteurized egg white powder in 50 children with moderate to severe allergic reactions to heated egg as diagnosed by double-blind, placebo-controlled food challenge. The successfully consumed egg dose increased in 90% of the children. After 8 months of OIT, 44% were desensitized to the target dose of 1 g, and 46% were partially desensitized to a median dose of 300 mg of egg white protein.

In this study, we examined humoral responses and profiled the blood cell transcriptome of these 50 children receiving egg OIT. We obtained plasma and PBMC samples at baseline and after 3 and 8 months of OIT, respectively. We found an extensive increase in Gal d 1–4-specific IgG4 and IgA and a significant decrease in IgE antibodies in their plasma samples as reported in our previous study (11). These results are in line with previous studies, where allergen desensitization and development of tolerance to foods were linked to the enhanced production of IgG4 and IgA, respectively (19, 20). To study allergic inflammation, we measured plasma cytokine responses and found that IL-1RA, IL-6, IL-17, and TNF decreased significantly throughout the OIT period. This suggests the attenuation of systemic inflammatory responses, which are believed to be maintained by the *de novo* production of leukotrienes, platelet-activating factor, and cytokines in the target organ (6).

In addition to the changes observed in the CD4/CD8 T cells and B cells, we evaluated OIT-driven changes with the transcriptome-based cell deconvolution method. The number of gamma/delta (γ/δ) T cells increased during OIT, whereas the number of circulating DCs and mast cells constantly decreased. Accumulating evidence demonstrates that γ/δ T cells play a critical role in regulating IgE responses. Different experimental systems suggest both IgE-enhancing and IgE-suppressive effects, though studies in humans are still lacking (21). DCs orchestrate and fine-tune most of the immunologic responses, and mast cells are the major source of allergen-specific IgE in the small intestine, ready to quickly release the mediators leading to allergic symptoms (22). Since the progenitors of mast cells are known to migrate into the bloodstream, their reduction in circulation might reflect the situation in the gut and other target tissues.

The PBMC transcriptome formed clusters both according to the sample collection time and gene functions. The differentially expressed genes of samples taken after 8 months of OIT enriched to innate immunity and defense response pathways accompanied by the downregulation of various metabolic processes and responses to stimuli (cluster III). Based on the biological processes of the GO terms, this might be due to changes in cell–cell adhesions of leukocytes, which exhibited the strongest fold enrichment in the pathways found. The transcriptomes formed also three patient clusters, of which the 8-month cluster was split into two according to the specific collection time after the beginning of OIT. The smaller cluster of 8-month samples might consist of patients whose responses have recently started to shift towards desensitization, whereas the larger cluster of the 8-month samples might consist of patients who have already reached tolerance because their gene expression pattern is very specific, especially in DEG clusters III and IV. After 3 months of OIT, we noticed a significant upregulation of a set of genes, including mainly long non-coding targets with unknown function that resolved by 8 months of OIT. It has been suggested that these long non-coding gene products play an important role in the regulation of gene transcription (23–25).

After 8 months of OIT, the majority of the enriched pathways had a negative activation score and were composed of downregulated genes playing a role in inflammation (IL-6, IL-8, IL-17, and AHR), acute phase responses, and Th2 pathway. The previous clinical findings support these gene expression reductions showing attenuation of pro-inflammatory cytokine production during an OIT (26, 27). Interestingly, the expression of nearly identical pathways was oppositely enhanced in another study, where PBMCs from egg-allergic patients were stimulated with egg extract (28). Upon stimulation, cells from egg-allergic individuals showed a strong enhancement in pathways of inflammatory responses, suggested to be transmitted through TNF and IL-6 signaling (28). A study describing the early transcriptomic effects of subcutaneous immunotherapy to grass pollen showed significant changes in the expression of TNF, IL-4, IL-10, TLR4, IL-27, and IFN-γ. (29) This suggests that opposite immunologic responses occur during allergen desensitization and allergen-specific stimulation of PBMCs.

Correlation analyses of antibody production and gene expression during OIT showed that an increase in Gal d 1–4-specific IgG4 antibodies during OIT was positively associated with innate immune responses and negatively with inflammatory signaling/responses. On the contrary, Gal d 1–4-specific IgE antibodies did not show strong correlations, suggesting that IgE is not associated with these gene functions. It is proposed that allergens interact directly or indirectly with innate PRRs, including toll-like receptors, and modify innate responses, especially during the development of allergic diseases (30). These allergen–PRR interactions could induce a NF-κB-dependent expression of proinflammatory cytokines or chemokines, such as IL-6, TNF, and type I interferons, which activate innate immune pathways with subsequent effects on adaptive immune responses (31). In addition to the PRRs, the increase of IgG4 was positively associated with a crosstalk between DC and NK cells and signaling of TREM1, which is an amplifier of the innate immune responses and a critical regulator of signaling during inflammation (32, 33). A recent study describing the transcriptomic profiling of PBMCs from subjects with food protein-induced enterocolitis syndrome before and after an oral food challenge found many common pathways and upstream regulators (*e*.*g*., TNF, LPS, IL-6, and TREM1) with our study (34). Therefore, we can postulate that food proteins or endogenous TLR ligands may induce the aberrant activation of PRRs and trigger the development of disease-associated gene expression patterns in susceptible subjects.

We did not discover any known functions for the DEGs upregulated in the 3-month samples nor were we able to cluster the partially (Part) or fully desensitized (Des) patients based on their gene expression profiles. However, when we studied humoral responses, the Part patients seemed to produce higher concentrations of antibodies and much less cytokines than the Des patients did. Especially the concentration of IgE antibodies to Gal d1–Gal d4 predicted the effectiveness of the OIT according to our dose–response regression studies. To find an explanation for these trends, we compared the gene expression of Part or Des to their own gene expressions at the beginning of OIT (0 months) and used a slightly looser threshold for the DEG analysis (*p*-value ≤0.05 and FC ≥|0.33|). This allowed us to also study less dramatic changes in gene expression, which still might have very important biological functions. It showed that Part patients still had Th2-prone responses at 3 months of OIT, and no upstream regulators were activated yet. At the same time, the DEGs of Des patients showed highly enhanced cellular metabolism, including energy transfer (oxidative phosphorylation), IL-10 signaling, and death-associated protein-3 (DAP3) upstream regulation. DAP3 mediates IFN-γ-induced cell death and is strongly associated with asthma, especially in patients with high serum IgE (35). At 8 months after OIT, the Part patients had enhanced antiviral response and inhibition of T cell activity (iNOS), while the Des patients showed enhanced activation of NK cells and Th1 responses. Natural killer (NK) cells are important factors in innate immunity (especially against viruses) that retain effector subsets, such as NK1 and NK2, with distinct cytokine profiles similar to Th1 and Th2-cells (36). However, a subset of NK cells display regulatory functions that suppress antigen-specific T cell responses *via* the secretion of cytokines (TGF-β and IL-10) and cell-contact-dependent mechanisms (37). Recent studies support the role of NK cell subsets in allergic diseases contributing to allergen-specific immune suppression, allergen-specific Th1 cell generation as well as IgE and other Ig production (38–40). Our transcriptomic data suggests that NK regulatory cells are activated during OIT not only in Des but also in Part patients. Although further studies are needed to link these cellular and humoral responses together, our findings suggest that NK cells are involved in the regulation of allergen-specific responses during OIT.

In summary, our study shows that egg OIT enhances innate immune and defense responses and attenuates various metabolic processes and responses to stimuli in PBMCs of egg-allergic children. During OIT, genes involved in the signaling of TREM1, IL-6, and IL-17 are downregulated, and the production of inflammatory mediators is decreased. This is accompanied by the increased production of allergen-specific IgG4 which correlates positively with pathogen recognition and the function of antigen-presenting cells suggesting that OIT modifies both adaptive immunity and innate immune responses. During the first phase of OIT, the expression of long non-coding genes with unknown biological functions increases, which may have an important role in the regulation of gene transcription. Then, in the later phase, allergen-specific cellular and humoral responses are inhibited, leading to the inhibition of inflammation and allergen-specific immune responses. We also show that very similar transcriptomic changes occur in all patients, but the changes are slower in individuals who are only partially desensitized after 8 months of OIT compared to the desensitized patients. Similar changes occur in both desensitized and partially desensitized patients, suggesting that successful desensitization may be achieved with prolonged treatment in most patients. Especially the desensitized patients showed signs of enhanced Th1 responses and activation of the suppressive functions of NK cells. This suggests that NK cells may regulate innate and adaptive immune responses and play an important role in immune homeostasis during food allergies and OIT. The increased understanding of the activated and deactivated pathways during OIT for food allergy facilitates the development of personalized treatment protocols and potential biomarkers for predicting the outcome of OIT.

## Data Availability Statement

The datasets presented in this study can be found in online repositories. The names of the repository/repositories and accession number(s) can be found below: www.ncbi.nlm.nih.gov, GSE178460.

## Ethics Statement

The studies involving human participants were reviewed and approved by the Helsinki University Hospital of Children and Adolescents Ethics Committee. Written informed consent to participate in this study was provided by the legal guardian/next of kin of the participants.

## Author Contributions

PK, TS, VH, and NF conceived the cell and plasma samples of this study. KP recruited all the patients and, in collaboration with MM, managed all the clinical aspects of the project. PK acquired and processed the array data and analyzed it in collaboration with HA. LW performed the leukocyte deconvolution estimations. PK and HA designed and drafted the figures. PK, KP, HA, and MM wrote the first draft of the paper, following discussion with and contributions from all authors. All authors contributed to the article and approved the submitted version.

## Funding

This work was supported by the Helsinki University Hospital Research Fund (grant no. TYH2019313 and TYH2020322), the Allergy Research Foundation, the Finnish Society of Allergology and Immunology, the Pediatric Research Foundation (grant no. 190150), the Sigrid Jusélius Foundation, Magnus Ehrnrooth foundation, the Finnish Cultural Foundation, and the Academy of Finland (grant no. 338325).

## Conflict of Interest

The authors declare that the research was conducted in the absence of any commercial or financial relationships that could be construed as a potential conflict of interest.

## Publisher’s Note

All claims expressed in this article are solely those of the authors and do not necessarily represent those of their affiliated organizations, or those of the publisher, the editors and the reviewers. Any product that may be evaluated in this article, or claim that may be made by its manufacturer, is not guaranteed or endorsed by the publisher.

## Abbreviations

DEG, differentially expressed gene; FDR, false discovery rate; OIT, oral immunotherapy; PBMCs, peripheral blood mononuclear cells; PRR, pattern recognition receptor; PTM, posttranslational modification.
