# Supplementary figures and images for: Integrative Transcriptomics Reveals Activation of Innate Immune Responses and Inhibition of Inflammation During Oral Immunotherapy for Egg Allergy in Children
Source: Front Immunol. 2021 Dec 15;12:704633. doi: 10.3389/fimmu.2021.704633 (PMC8714802; doi:10.3389/fimmu.2021.704633)

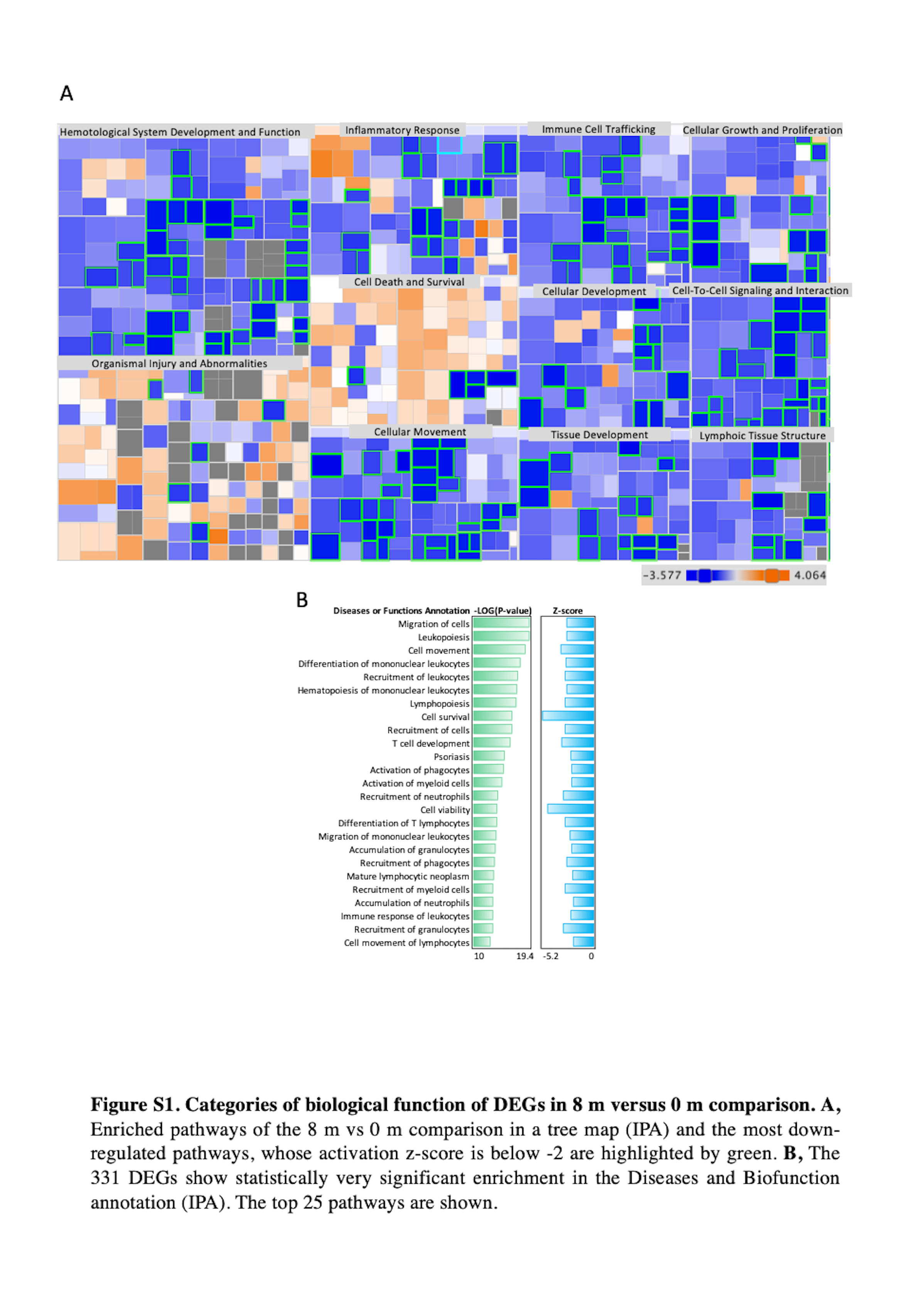

Supplement: Supplementary file 1 [file Image_1.jpeg]

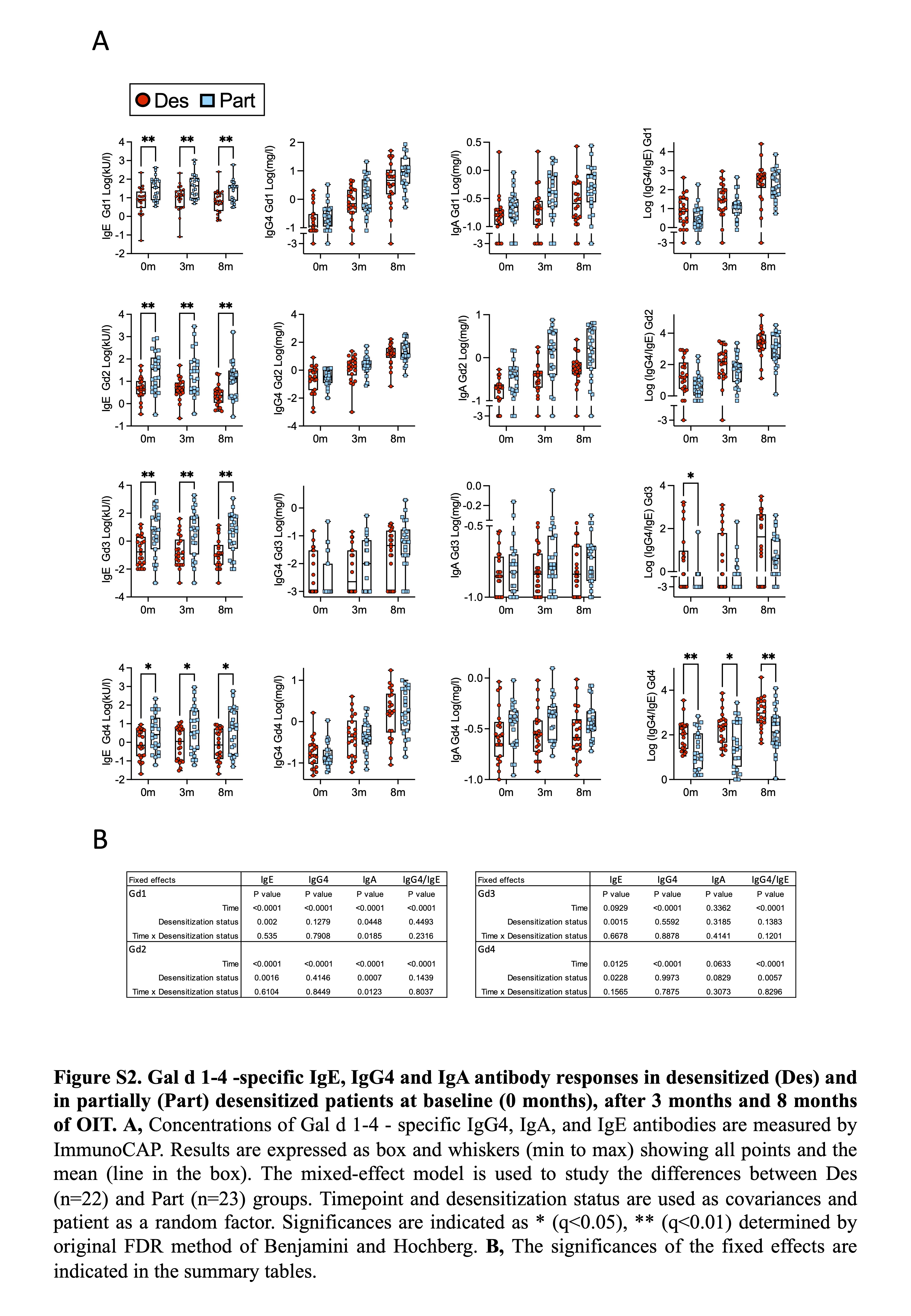

Supplement: Supplementary file 2 [file Image_2.jpeg]

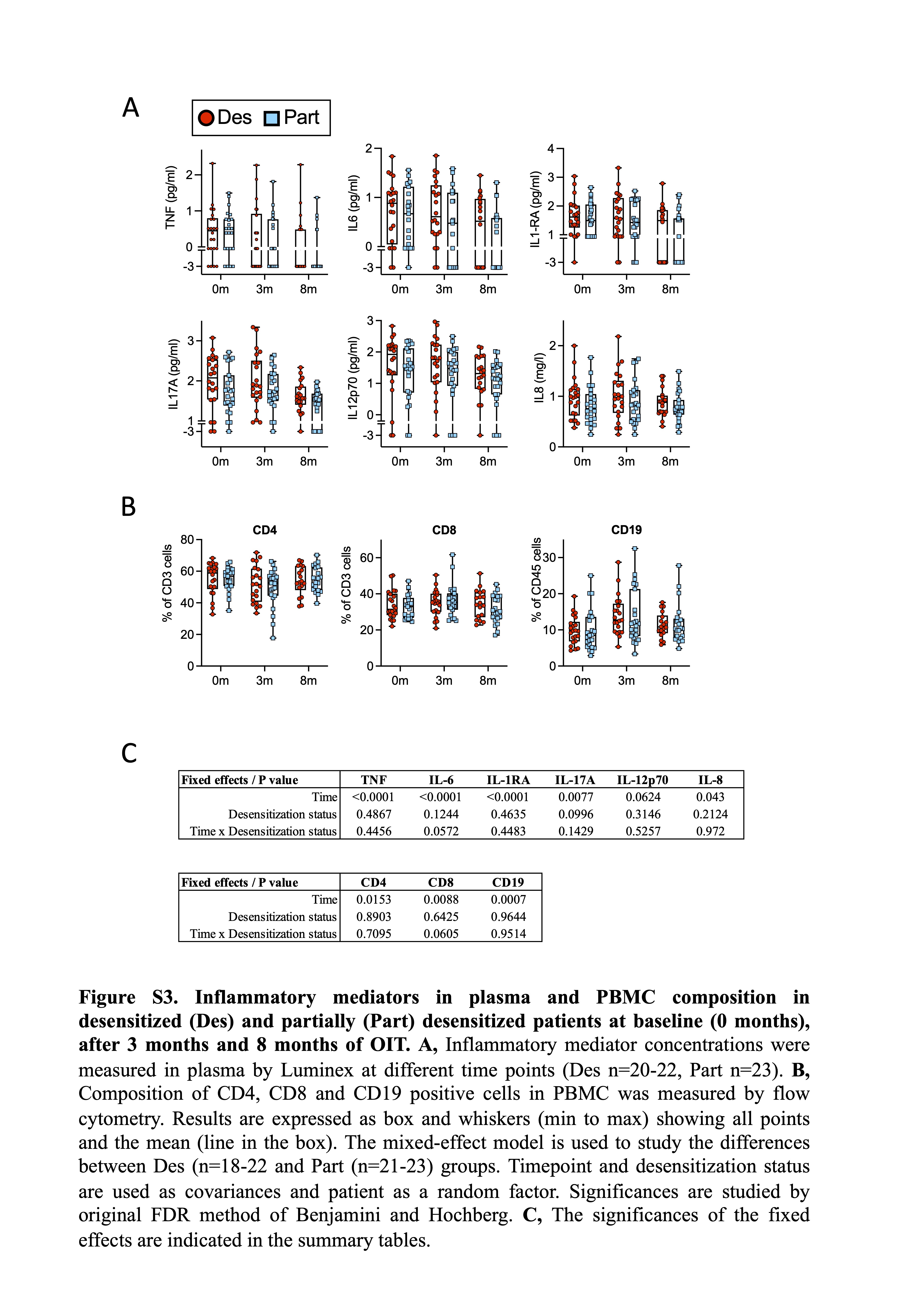

Supplement: Supplementary file 3 [file Image_3.jpeg]

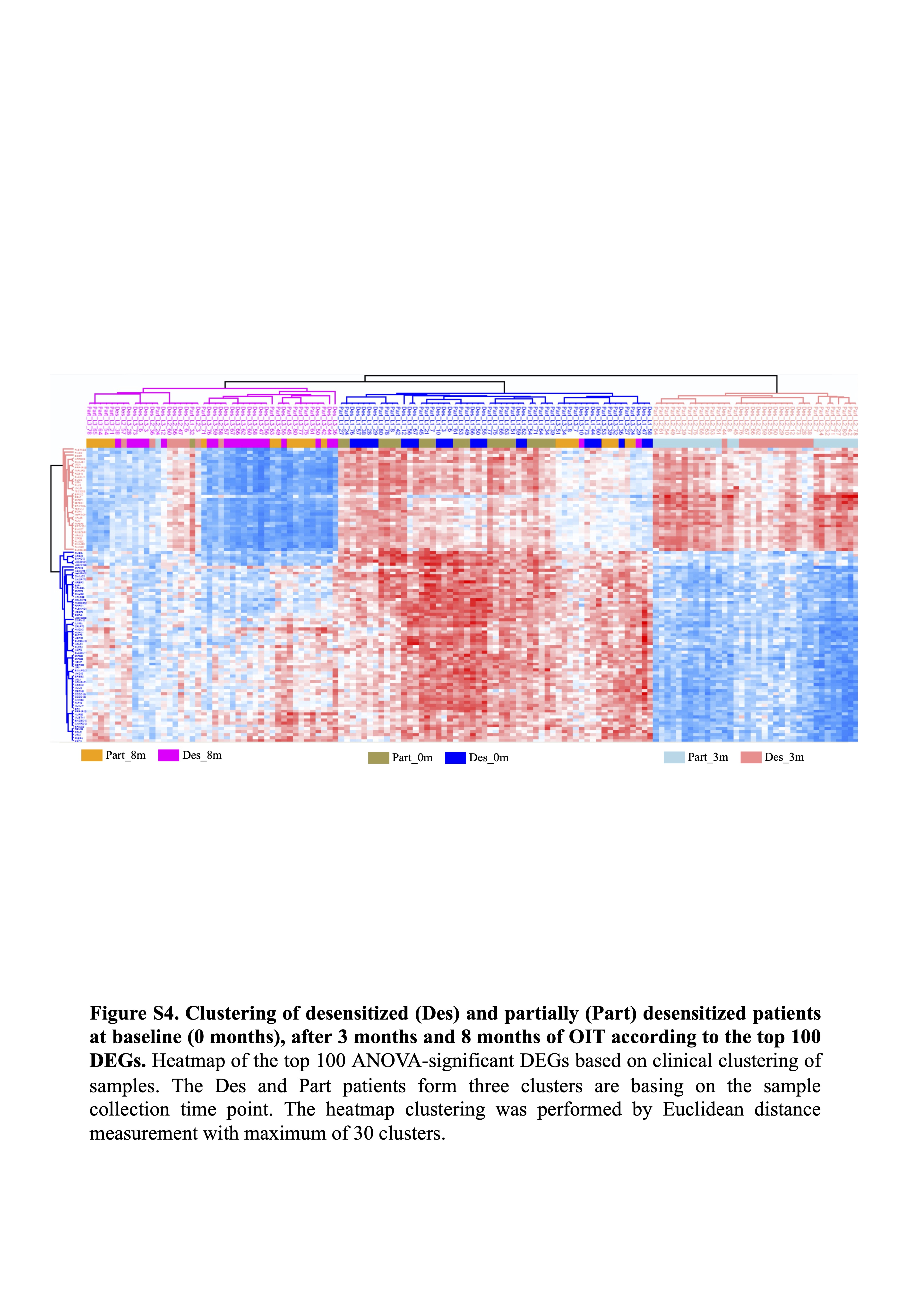

Supplement: Supplementary file 4 [file Image_4.jpeg]

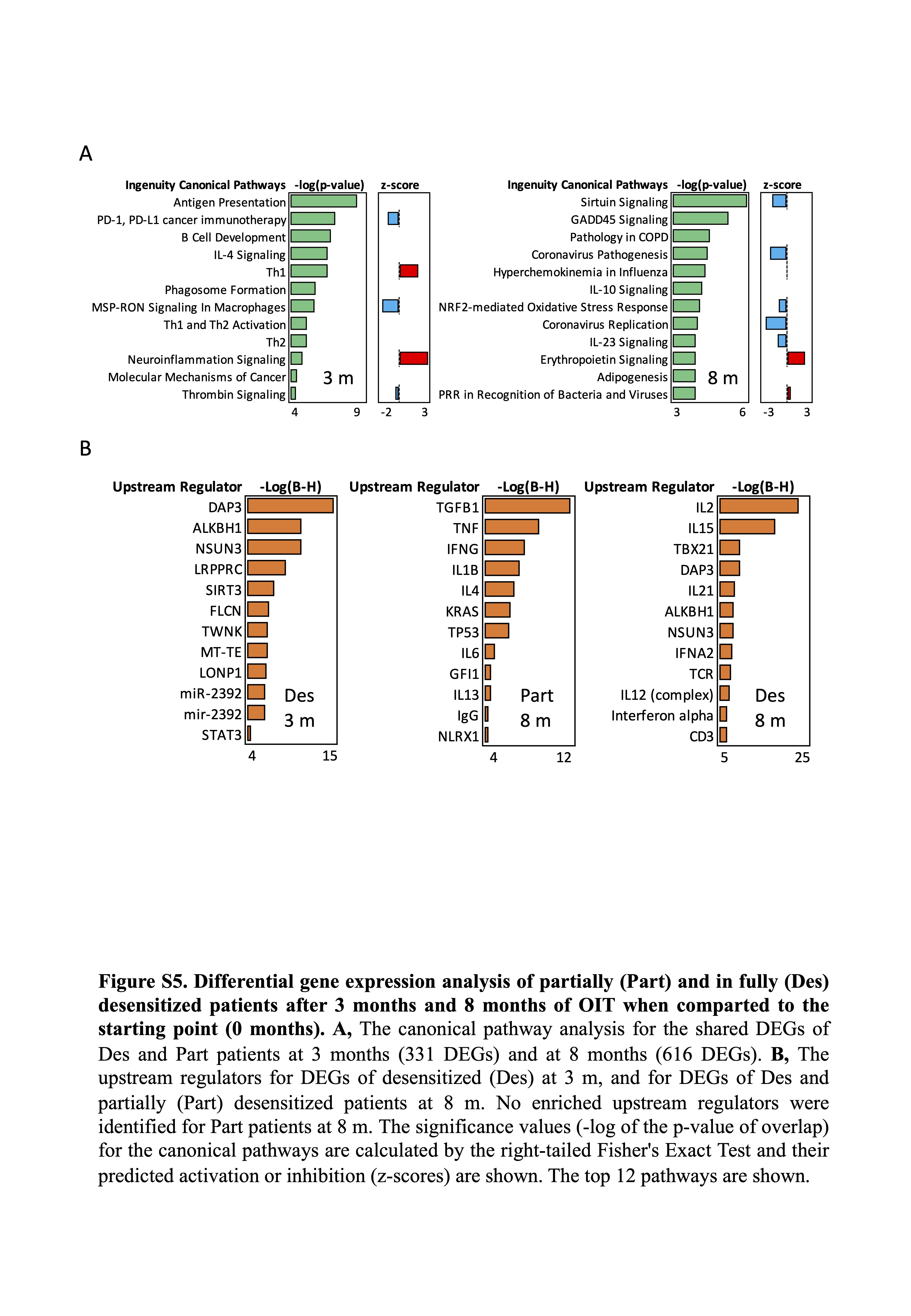

Supplement: Supplementary file 5 [file Image_5.jpeg]

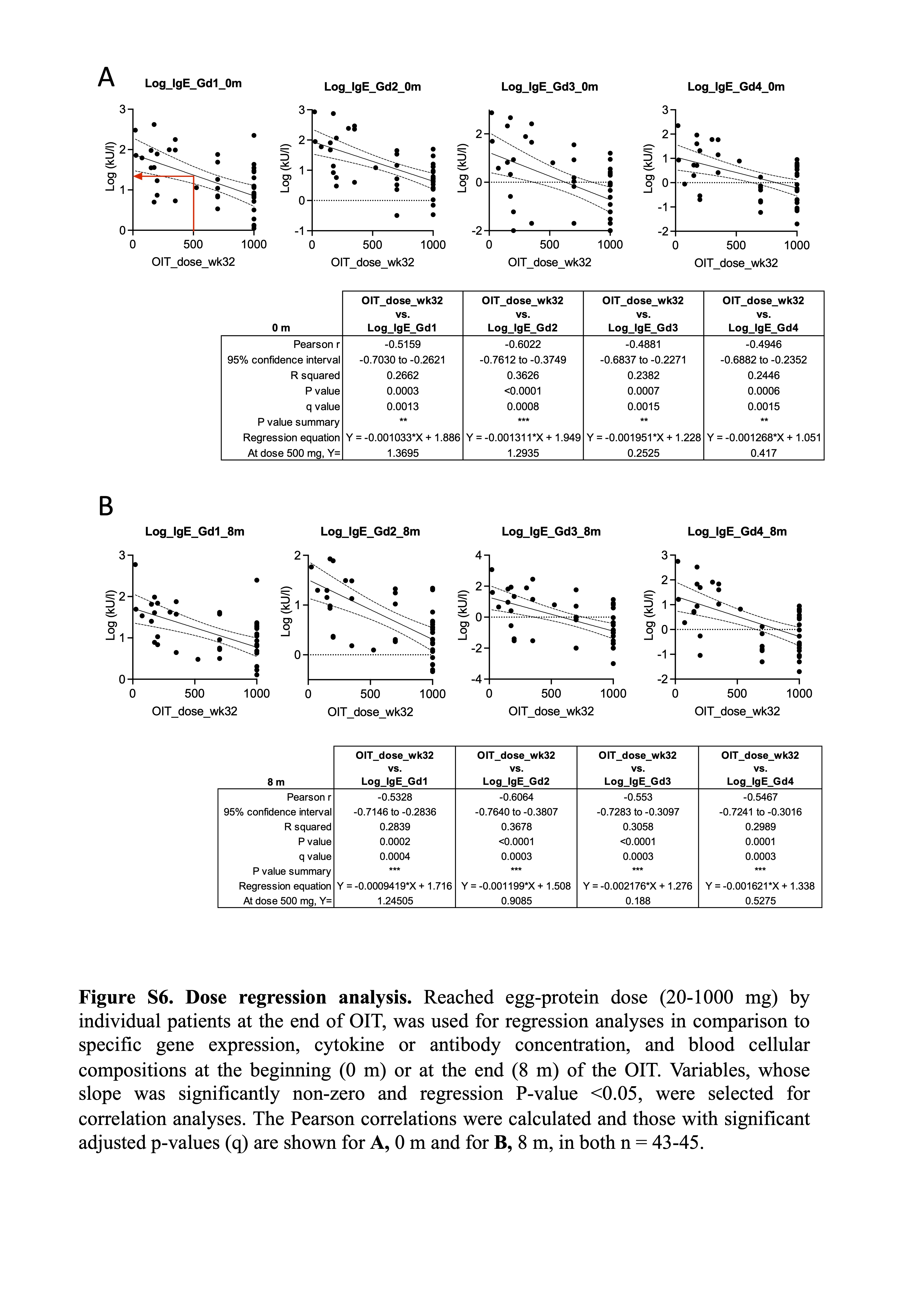

Supplement: Supplementary file 6 [file Image_6.jpeg]
